# Protocol to analyze marrow B lineage cell dynamics by *in vivo* three-photon microscopy in intact mouse tibia

**DOI:** 10.1016/j.xpro.2025.103824

**Published:** 2025-05-15

**Authors:** Anne Bias, Alexander F. Fiedler, Robert Günther, Ruth Leben, Ingeborg Beckers, Anja E. Hauser, Asylkhan Rakhymzhan, Raluca A. Niesner

**Affiliations:** 1 Dynamic and Functional *in vivo* Imaging, Freie Universität Berlin, 14163 Berlin, Germany; 2 Biophysical Analytics, German Rheumatology Research Center – a Leibniz Institute, 10117 Berlin, Germany; 3 Medical Physics/Physical Engineering, Berlin University of Applied Science and Technologies, Berlin, Germany; 4 Rheumatology and Clinical Immunology, Charité – Universitätsmedizin, Berlin, corporate member of Freie Universität Berlin and Humboldt-Universität zu Berlin, 10117 Berlin, Germany; 5 Immune Dynamics, German Rheumatology Research Center – a Leibniz Institute, 10117 Berlin, Germany; 6 Department of Fundamental Medicine, Higher School of Medicine, Al-Farabi Kazakh National University, Almaty 050040, Kazakhstan

**Keywords:** Biophysics, Immunology, Microscopy

## Abstract

Three-photon microscopy (3PM) allows deep-tissue imaging beyond the capabilities of two-photon microscopy (2PM) owing to infrared excitation. Here, we present a protocol for time-lapse 3D imaging of B lymphocytes in the tibia marrow of fluorescent reporter mice using 3PM at 1,650 nm. We describe steps for verifying microscope performance and tibia imaging. We then detail the cell dynamics analysis, including denoising, cell segmentation, and tracking. This protocol has potential application for immune cell tracking in other optically inaccessible organs in which 2PM fails.

For complete details on the use and execution of this protocol, please refer to Rakhymzhan et al.^1^

## Highlights

• Guidance on three-photon microscopy performance verification at 1,650 nm
• Steps for optimized time-lapse three-photon deep-marrow imaging in intact tibia
• Procedure for marrow B lymphocyte dynamics analysis in unperturbed marrow
• Instructions on machine-learning-based denoising for cell segmentation and tracking

## Abstract (editor highlights)

Publisher’s note: Undertaking any experimental protocol requires adherence to local institutional guidelines for laboratory safety and ethics.

## Graphical abstract

**Figure:**
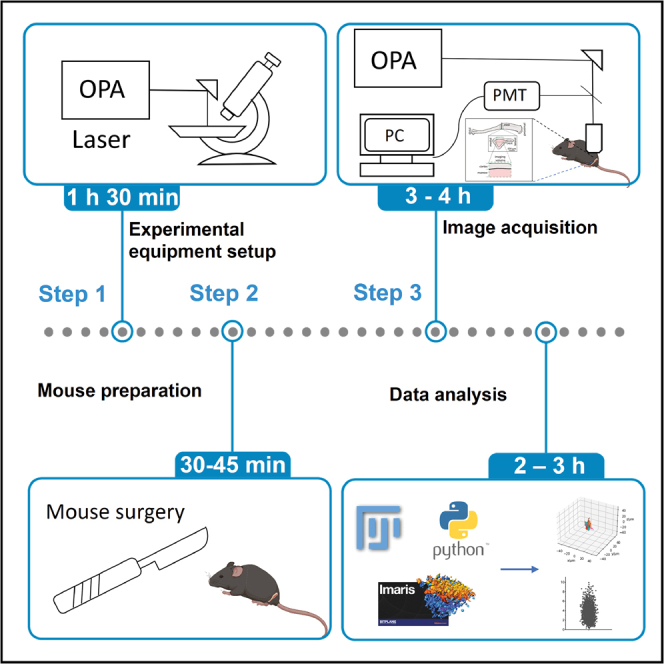

## Before you begin

Three-photon microscopy (3PM) allows *in vivo* deep-tissue imaging of optically hardly accessible organs, beyond the capabilities of two-photon microscopy (2PM) due to long wavelength excitation, either in NIR I (1300 nm) or NIR II (1700 nm) tissue windows.^2^^,^^3^^,^^4^ Several improvements of the optical system, including beam shaping,^5^ adaptive optics,^6^^,^^7^^,^^8^^,^^9^^,^^10^^,^^11^ the design of new objective lens concepts,^12^ as well as the development of dedicated fluorophores are currently boosting the application of the technology.^13^ 3PM performance has been successfully demonstrated *in vivo* in brain cortex,^6^^,^^14^^,^^15^ deep tumor^16^ as well as soft lymphoid organs imaging.^17^

Due to strong scattering effects in calcified bone, perturbation-free *in vivo* imaging of the marrow cavity through the intact thick bone cortex in long bones of adult mice still poses a challenge. This strong scattering is related to the fact that bone has the highest refractive index span among mammalian tissues (from n ≈1.33 up to ≈1.62),^18^^,^^19^^,^^20^ for comparison, the refractive index of soft lymphoid tissues ranges between ≈1.33 and ≈1.4.^18^^,^^21^ The importance of bone marrow imaging can hardly be overestimated: as primary lymphoid tissue, the bone marrow is the birthplace of most immune cells,^22^ including B cells, and the final destination of terminally differentiated cells ensuring long-term immunological memory, such as plasma cells.^23^

We previously described the implementation of an optical parametric amplifier prototype emitting at 1650 nm with a variable repetition rate between 1 and 4 MHz in 3PM and demonstrated its performance for *in vivo* imaging of the tibia marrow through the intact bone cortex in mice.^1^ In this way, we were able to perform time-lapse imaging over large imaging volumes (400 × 400 × 30 μm^3^) in CD19:LSL-tdRFP fate mapping mice and to analyze the dynamics of B lymphocytes, without inducing tissue photo damage. We could analyze the motility of sufficient numbers of both highly abundant B cells and rare plasma cells, ensuring reliable statistical analysis and linking plasma cell motility patterns to functional state, based on third-harmonics generation (THG) signals of cellular organelles.

Here we describe a protocol to perform reliable three-photon imaging in the marrow of intact mouse tibia, including a routine verification procedure of the microscope performance, and to analyze B lymphocyte dynamics from the generated time-lapse 3D imaging data. While the described protocol refers to marrow B lineage cells imaging in intact tibia, it is applicable to other immune cell types, also in flat bones, i.e. calvarium, as well as in secondary lymphoid organs, i.e. lymph node and spleen. Three-photon microscopy in the calvarium,^24^ i.e. bone with a low water content, is favorable at 1650 nm, due to lower scattering at this wavelength as compared to 1300 nm.^21^ In soft tissues with higher water content, such as lymph node and spleen tissues,^17^ three-photon microscopy is more efficient at 1300 nm, as at this wavelength the water absorption is lower than at 1650 nm.^21^

### Institutional permissions

All animal experiments were conducted in accordance with the ARRIVE guidelines and approved by Landesamt für Gesundheit und Soziales, Berlin, Germany in accordance with institutional, state and federal guidelines (G0048/21). Adult female and male mice (12-24 weeks old) were randomly assigned to experimental groups, as it is known that male mice have thicker cortical bone than females. The mice exhibited healthy behavior and weight and were housed in a conventional pathogen-free SPF barrier facility with enriched and suitable cage spaces with drinking water and a standard chow diet ad libitum as recommended by the authorities.

To perform the animal experiments described in the protocol, prior approval according to the institutional or state legislation is required.

### Verification of optical performance of the three-photon microscope

**Timing: 1 h 30 min**

1. Prepare a sample of fluorescent nanospheres embedded in agarose for imaging.
  a. Prepare a suspension of fluorescent nanospheres of 100 nm diameter by diluting 1:1000 the 2% stock solution (λ_em_ = 605 nm) and pipette 300 μL in a 1.5 mL Eppendorf tube.
  b. Heat up a mixture of 2% low-melting agarose in distilled water in an open beaker.
  c. While the agarose is still fluid, pipette 700 μL of the agarose suspension in the tube containing 300 μL nanospheres suspension and mix thoroughly.
  d. Pipette the still-fluid mixture of nanospheres in agarose on a glass slide and allow it to solidify.

**CRITICAL:** Keep the temperature moderate to avoid nanospheres damage and ensure building an agarose sample of at least 1 mm height.

**Figure 1:**
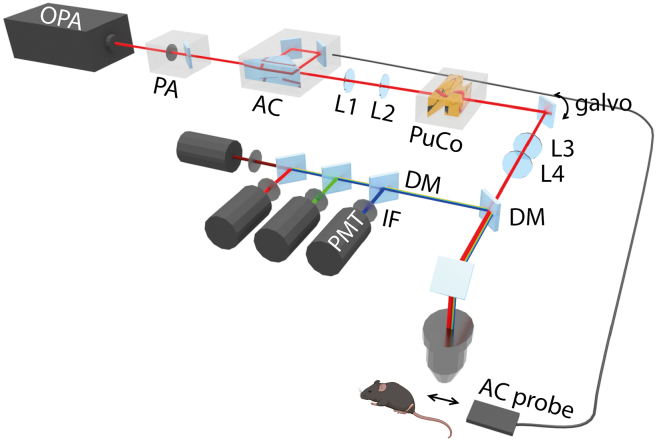
Schematics of the three-photon microscope OPA: Ytterbia laser emitting at 1650 nm, pulse width 65 fs, repetition rate 1,2,3 or 4 MHz; PA: power attenuator, based on a rotatable λ/2 plate and a fixed polarizer; AC: second-order interferometric autocorrelator, internal part; L1, L2: convex lenses building a beam expander; PuCo: pulse compressor containing a ZnSe wedge (adjustable position) and a fused silica wedge; galvo: x and y rotating galvanometric mirrors; L3: scan lens; L4: tube lens; DM: dichroic mirror with cut-off at 795 nm; objective lens (Olympus XPLN25XWMP2; 25×; multi-immersion; NA 1.05); DM and IF (detection): dichroic mirrors and interference filters to analyze the emitted radiation. PMT: photomultiplier tubes used as detectors. AC probe: external probe of the second-order interferometric autocorrelator, placed under the objective lens. For *in vivo* imaging, the anesthetized mouse with exposed and fixed tibia is placed under the microscope.

2. After turning on the laser and microscope components and starting the control software, align the beam path of the optical parametric amplifier to be centered on the back aperture of the objective lens (Figure 1).

**CRITICAL:** Use either IR indicator paper or an IR beam viewer suitable for 1650 nm to make the beam visible.

**CRITICAL:** Do not use the scan mode for alignment and set the parking position of the scanner mirrors to the default aperture center (0;0), i.e. the optical axis of the system.

3. Measure average laser power at laser output and under the objective lens using a power meter.

***Note:*** Ensure that the position of the λ/2 plate of the power attenuator is set to match the transmitted polarization by the thin-film polarizer (Figure 1).

***Note:*** The transmitted power should be at least 25% of the power at the laser output.

**CRITICAL:** Do not use the scan mode for alignment and set the parking position of the scanner mirrors to the default aperture center (0;0), i.e. the optical axis of the system.

4. Measure the autocorrelation function of the laser pulse train at the laser output using the internal mode of the autocorrelator by second-order interferometry (Figure 1).

**Figure 2:**
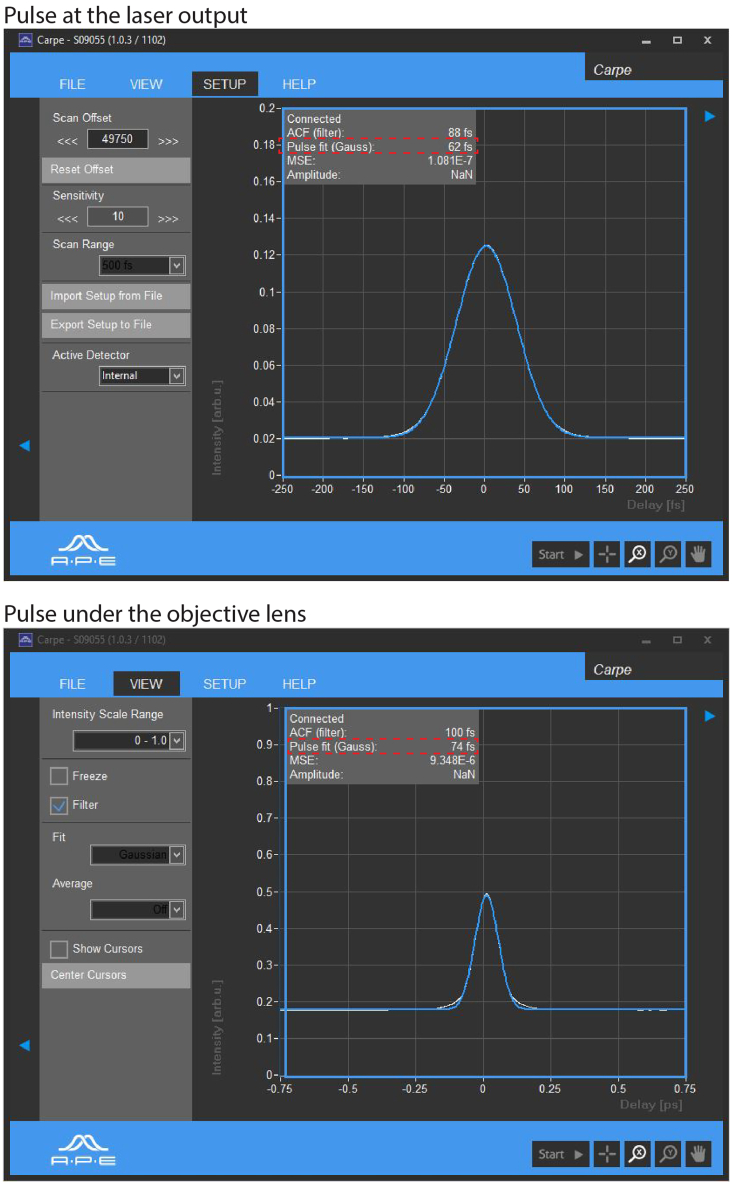
Pulse width analysis Temporal pulse width measurement of the 1650 nm laser beam at laser output and under the objective lens, by second-order interferometric autocorrelation. The pulse widths are shown in the second line marked by the dashed line.

***Note:*** Using the provided software routine of the CARPE a Gaussian or sech^2^ pulse is calculated and its width is displayed (Figure 2).

5. Measure the autocorrelation function of the laser pulse train under the objective lens using the external mode of the autocorrelator by focusing the laser beam on the detection area of the external probe (Figure 1).

***Note:*** The software displays also for the external measurement the fitted Gaussian or sech^2^ function. Choose the function directly in the software. Figure 2 shows Gaussian pulses.

6. While measuring the autocorrelation function externally, adjust the μm-screw position of the pulse compressor to vary the ZnSe thickness in the beam path, minimizing the pulse width.

***Note:*** With steps 4-6 the pulse width at the sample surface is minimized and photon flux density needed for three-photon processes is maximized at the given average laser power, reducing the risk of tissue photo damage.

**Figure 3:**
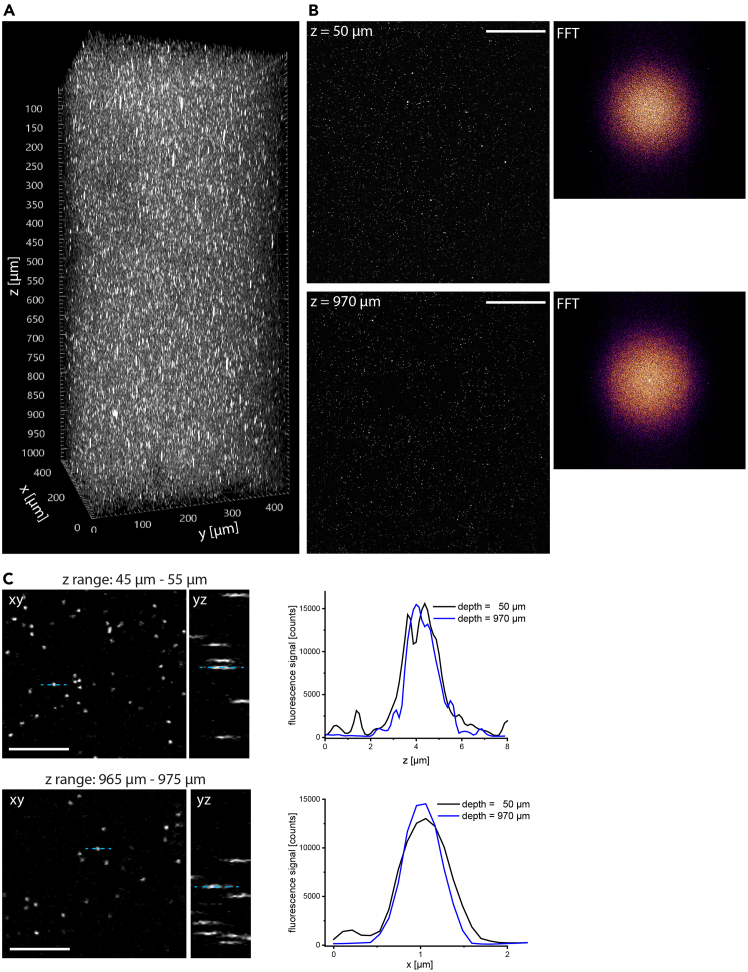
Three-photon microscope optical performance (A) 3D image (442 × 442 × 1031 μm^3^) of 100 nm fluorescent nanospheres embedded in agarose. (B) Slice images of the fluorescent nanospheres from A shown at the depth 50 μm and 970 μm and their Fourier-transformed (FFT) images, respectively. Scale bar: 100 μm. (C) xy- and yz-projections at the z-ranges 45-55 μm and 965-975 μm, respectively (left). Lateral (x) and axial (z) fluorescence signal profiles corresponding to the dashed blue lines in the left images (right). Scale bar: 20 μm.
  b. Upload both small 3D volume data in ImageJ/FIJI and generate x/y profiles as well as z profiles of single nanospheres both at the surface (45-55 μm depth) and deep (965-975 μm depth) in Figure 3C.
  c. Plot x/y profiles as well as z profiles for both depths to check for changes in profile shape and width, indicating a distorted wavefront. While we used Origin, MATLAB, Python or Excel can be used as well.
  d. Fit the x/y and z profiles by Gaussian functions to determine the width of the effective point spread function, as the 100 nm nanospheres are smaller than the diffraction limit of the system.
  e. Compare the values with theoretical lateral and axial resolution calculated based on the Debye vectorial approximation.
    ***Note:*** Steps 7 and 8 represent a quick way to diagnose coarse optical misalignment and wavefront distortions in the optical system.

7. Switch the controlling software of the microscope (ImSpector) to the scan mode (442 × 442 μm^2^, 1036 × 1036 pixel), moving the parking position outside of the scan lens aperture to block the laser beam when not in use.
  a. Place the glass slide with the agarose sample under the objective lens (Olympus, XLPLN25XWMP2, 25×, NA 1.05), ensuring water immersion.
  b. Acquire an overview 3D image of the fluorescent nanospheres embedded in agarose, detection channel 594±20 nm. Ensure oversampling for 1650 nm and NA = 1.05, i.e. a 442 × 442 × 1031 μm^3^ volume is acquired as 1036 × 1036 × 1031 voxel (xy-pixel = 426.64 nm, Δz = 1 μm).
  c. At two selected depths (at the surface, z between 45 and 55 μm, and deep, z between 965 and 975 μm) acquire 3D stacks of 50 × 50 × 10 μm^3^ volume (469 × 469 × 41 voxel; 106 nm xy pixel, Δz = 250 nm).
  d. Export 3D image data as tiff files.
8. Verifying the expected spatial resolution of the microscope.
  a. Upload large 3D volume data (442 × 442 × 1031 μm^3^) in ImageJ/FIJI (Volume Viewer) or Imaris for 3D reconstruction (Figure 3).
    ***Note:*** Inspect the 3D volume for deviations in intensity or elliptical shape of microsphere signal, both across the xy field of view and along the optical (z) axis, as these represent a hint towards optical misalignment.
    **CRITICAL:** In ImageJ/FIJI calculate the Fourier-transform of an xy image near the surface and one deep within the sample. Similar FFT (Fast Fourier Transform) images hint towards appropriate optical alignment (Figure 3B).

### Preparation of mouse tibia for *in vivo* imaging

**Timing: 30–45 min**

9. Mouse anesthesia.
  a. Holding the mouse (CD19:tdRFP fate mapping mouse strain) fixed, apply an inhalation mask on its nose.
    ***Note:*** For ventilation, use a mixture of oxygen and isoflurane (isoflurane concentration between 1.5 and 2%).
  b. Adjust the concentration of isoflurane and oxygen flow rate according to the weight of the mouse.
    ***Note:*** For a mouse mass between 20 g and 35 g, use flow rates between 200 mL/min and 500 mL/min.
10. Place the anesthetized mouse on a heated plate and keep the core temperature at 37°C, e.g. using a rectal thermometer.
11. Mouse surgery for imaging.
  a. Fix the right hind paw by stretching the leg to the side.
  b. To prevent contamination, shave the fur over the entire limb.
  c. Make an incision in the skin above the tibia.
  d. Push the tibialis anterior muscle aside, to expose the flat medial surface of the tibia shaft.
  e. To keep the tibia in position, fix the crest with a surgical hook.

**Figure 4:**
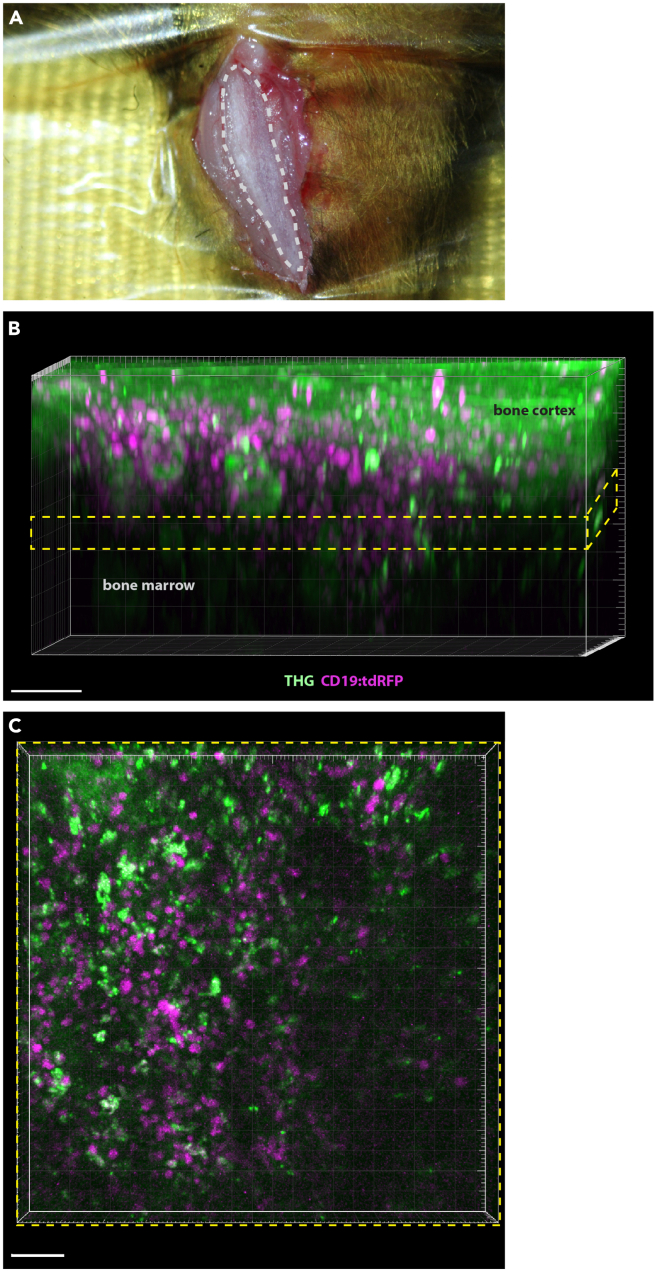
Three-photon imaging at 1650 nm (3 MHz repetition rate) in intact tibia of CD19:LSL-tdRFP mice (A) Picture of the mouse leg showing the tibia prepared for imaging, with the search area marked by the gray dashed line. (B) Rendering of an overview 3D image in the mouse tibia of a CD19:LSL-tdRFP mouse, used for orientation in tissue. Third harmonics generation (THG, green) and tdRFP fluorescence signal (magenta) in marrow (C) lineage cells allow to differentiate between bone cortex and bone marrow. Yellow dashed box indicates the volume defined for time-lapse imaging. (B) Rendering of the 3D volume (400 × 400 × 30 μm^3^) imaged in a time-lapse manner (time step = 30 s). Scale bar: 50 μm.

***Note:*** The tibial area in which imaging can be performed, i.e. search area of 10 mm^2^ (more than one third of the total anterior tibial length), is located on the anterior part of the tibia, next to the lower half of the crest (arch), as highlighted in Figure 4.

12. Preparation of the imaging site for using the water immersion objective lens.
  a. Build a low melting agarose wall (1% agarose) around the open area to form a cylindrical bath.
  b. Fill the agarose cone with isotonic NaCl solution as immersion medium for imaging.
  c. Fill up the still fluid agarose in a syringe (without a needle) to build the agarose wall layer by layer.

## Key resources table

**Table:** 

REAGENT or RESOURCE	SOURCE	IDENTIFIER
**Chemicals, peptides, and recombinant proteins**		

FluoSpheres carboxylate-modified nanospheres	Thermo Fisher Scientific	F8810
Low-melting agarose	Sigma-Aldrich	A4018
Isoflurane	Baxter	26675-46-7 (CAS-No)

**Deposited data**		

Imaging data – tibia	Zenodo	https://doi.org/10.5281/zenodo.8383833
Imaging data – nanospheres in agarose	Zenodo	https://doi.org/10.5281/zenodo.7464124

**Experimental models: Organisms/strains**		

Mouse strain: CD19:tdRFP:Cd19tm1(cre) ki/wt Gt(ROSA)26Sortm1Hjf dfl/dfl; age 8–10 weeks; males and females	Crossed at DRFZ	MGI No: 1931143 (CD19), 3696099 (tdRFP)

**Software and algorithms**		

Rose plot generation	GitHub	https://github.com/niesner-ra/Tracks
Trained N2V algorithm	GitHub	https://github.com/niesner-ra/N2V_3PM
Origin Pro	OriginLab	https://www.originlab.com/
Python version 3.9	Python	https://www.python.org/
Fiji	Fiji	https://fiji.sc/
Imaris version 9.7.2	Oxford Instruments	https://imaris.oxinst.com/
ImSpector	Miltenyi Biotec	N/A

**Other**		

Optical parametric amplifier (Ytterbia, 1,650 nm, 60 nm band width, 65 fs pulse width, repetition rates 1.01, 2.06, 3.01, 3.98 MHz)	Thorlabs Inc Laser Division, CO	FSLOPAX1
TrimScope II upright microscope	Miltenyi Biotec GmbH (former LaVision Biotec)	TrimScope II/Matrix
Photomultiplier tubes, H7422-40 and H7422-50	Hamamatsu, Japan	H7422-40 H7422-50
Objective lens, XPLN25XWMP2, 25×, multi-immersion, NA 1.05	Olympus (Evident), Hamburg	XPLN25XWMP2
CARPE (interferometric autocorrelator; AR coating for the range 1,200–1,700 nm)	APE, Berlin	CARPE IR
Power meter	Newport, Duisburg	843-R-USB
IR indicator card, 800–1,700 nm	Thorlabs, CO	VRC2

## Step-by-step method details

### Time-lapse image acquisition of B lymphocytes in the tibia of CD19:LSL-tdRFP mice

**Timing: 3–4 h**

Following the steps in this section, three-dimensional tdRFP fluorescence specific for marrow B lymphocytes, second-harmonics and third-harmonics images acquisition over time will be accomplished.

1. Getting started for imaging.
  a. Place the heated plate with the anaesthetized mouse on the microscope stage.
  b. Immerse the objective lens in the isotonic NaCl solution.

***Note:*** Ventilation should be controlled continuously.

2. Preparing the microscope setup and room for the imaging experiment.
  a. Adjust the z position of the objective lens until you see through the ocular the tibia surface.
  b. Darken the room.
3. Searching for the desired anatomical site and optimal microscope settings for imaging.
  a. In the acquisition software, start video recording in the scan mode (full field of view, FOV, 442 × 442 μm^2^) to find the bone cortex (in the THG channel detected at 562 ± 20 nm osteocytes are a good indicator, Figure 4).
  b. Use a line frequency of 400 Hz and no frame averaging, to ensure fast acquisition required for time-lapse imaging.
  c. Scan the tissue along the z-axis to surpass the bone cortex and reach the bone marrow.
  d. Increase the average laser power as you move the focus deeper into the tissue, from *P*_*0*_ = 2 mW at the surface (z = 0 μm) to 32 mW at z = 300 μm depth.
  e. Follow an exponential power increase *P(z)* with depth (z in μm), with an increase rate *k* of 0.0092 μm^−1^ ($P\left(z\right)=P_{0}\cdot e^{k\cdot z})$
  f. Use z-step intervals of 2 μm.
4. Large 3D stack acquisition for anatomical orientation in tibia.
  a. Record a large volume 3D stack (442 × 442 × 300 μm^3^) encompassing both bone cortex layers and marrow (Figure 4).
  b. Use exponential z-adaptation of laser power, as the effective attenuation of radiation in bone is expected to be high.
  c. Record both tdRFP fluorescence in B lymphocytes (detection at 594 ± 20 nm) and THG signal (detection at 562 ± 20 nm).

**CRITICAL:** Excitation at 1650 nm enables 3PM imaging of marrow cells through up to 200 μm thick cortical bone, which is not possible at shorter excitation wavelengths, neither by 3PM (at 1300 nm) nor 2PM (at 1100 nm).^1^

5. Selecting the 3D region for time-lapse imaging.
  a. Within this 3D stack, select the boundaries of a 30 μm thick layer in the bone marrow (400 × 400 × 30 μm^3^, 517 × 517 × 11 voxel), defining the 3D volume for time-lapse imaging.
  b. Ensure that the acquisition time of the 3D stack is less than the time-step chosen for time-lapse imaging.
  c. Adjust line frequency (400 Hz) and average power to control the fluorescence signal.
  d. Keep the average laser power to a minimum to avoid tissue photo damage and photobleaching.
6. Record the time-lapse 3D stacks over a duration of **1 h** with intervals of **30 s.**

***Note:*** For longer acquisition time windows, e.g. **2 h** or more, increase the time intervals to **120 s**.

**CRITICAL:** Control the organ preparation stability as detailed in the “troubleshooting” section. Re-adjust the desired position and re-start measurement, if the position of the imaged area changes over time.

### Analysis of B lymphocyte dynamics in time-lapse 3PM data of tibia marrow

**Timing: 2 h**

This section accomplishes the analysis of B lymphocyte dynamics in the tibia marrow from the time-lapse 3D imaging data acquired by three-photon microscopy at 1650 nm, relying on image de-noising, cell segmentation and tracking. Further, statistical analysis of mean cell velocity, mean displacement rate as well as of cell volume to resolve between naïve B cells and plasma cells is described.

**Figure 5:**
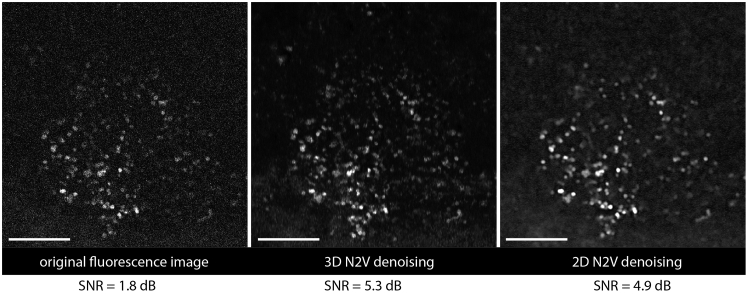
Denoising of tdRFP fluorescence signal in the tibia marrow Original tdRFP fluorescence image (left) acquired in the marrow of the intact tibia of a CD19:LSL-tdRFP mouse by three-photon microscopy. Signal to noise ratio (SNR) is 1.8 dB. Denoising performance of Noise2Void (N2V) trained on 3D data (middle, SNR = 5.3) and on 2D data (right, SNR = 4.9). SNR = 10·lg(<I_signal_>/σ_signal_), with <I_signal_> mean signal intensity and σ_signal_ standard deviation of signal in the image. Scale bar: 100 μm.

***Note:*** For N2V training we recommend the following setting: 20 images for each run, 200 epochs, 300 steps/epoch, 128/256 batch size per step, and neighborhood radius 5.

7. Training the deep-learning model with own data for image denoising.
  a. To improve the signal-to-noise ratio (SNR) in the acquired data, train Noise2Void (N2V) plug-in, the open-source neural network algorithms CSBDeep package in FIJI on high SNR 3D stacks of B cells in the tibia marrow.
    ***Note:*** The trainable deep-learning algorithm N2V allows to predict original cell shapes without clean targets.
  b. Perform training on high SNR imaging data, e.g. acquired in the tibia marrow through mechanically thinned bone cortex.
  c. Use for comparison both 2D data and 3D data, acquired in different tissue depths, in different experiments.
8. Validate the model on high SNR data not involved in the training process and on low SNR data acquired through intact bone (Figure 5).

**CRITICAL:** Perform training for each detection channel individually.

9. Apply the trained N2V model to the time-lapse 3D imaging data.

***Note:*** The fluorescence signal in each pixel is restored by reducing the estimated noise, whose distribution differs in a certain neighborhood, known as the receptive field. De-noising facilitates cell segmentation and tracking.

10. Getting started with cell dynamics analysis.
  a. Upload the de-noised time-lapse 3D imaging data displaying tdRFP fluorescence in B lineage cells in Imaris.
  b. Adjust geometry parameters according to the image acquisition parameters and image contrast for best visualization.

**CRITICAL:** The correct spatial dimensions in μm and time dimension in seconds are key to ensuring correct cell motility analysis.

11. In Imaris, create a Surface reconstruction for cell segmentation and tracking.
  a. Set the surface roughness at 1 μm and use local contrast for the Watershed algorithm, with the largest sphere fitting within the object set to 7 μm (the estimated dimension of a B cell).
  b. Split objects based on an estimated average size of 10 μm.
  c. Exclude objects smaller than 65 μm^3^ (diameter < 5 μm, cell debris) and larger than 4096 μm^3^ (diameter > 20 μm).
  d. After objects have been identified at all time points, perform tracking using the Autoregressive Motion algorithm, with an estimate maximum distance between consecutive time points of 15 μm and a maximum gap size of 1 gap.
  e. Visually inspect the tracks to exclude numerical artifacts of the automated tracking.
  f. Merge tracks or split tracks manually to correct these artifacts.
12. Resolving and characterizing B and plasma cells in the analyzed images.
  a. In the “Surface” environment on Imaris, define two object groups by cell volume: one group with volumes between 65 μm^3^ and 500 μm^3^, i.e. B cells, and another group with volumes between 500 μm^3^ and 4100 μm^3^, i.e. plasma cells.
  b. Extract statistics, i.e. individual cell trajectories, displacements, track lengths and track durations and export these as a csv or txt file, or as individual files of the same type.
13. Graphical representation of B and plasma cells’ trajectories.
  a. Import the statistics file containing only cell trajectories for both B and plasma cell populations in the Python routine to generate rose plots.

***Note:*** Rose plots are graphical representations of object trajectories, all with the origin in (0,0,0) of the Cartesian coordinate system. The code you can use, therefore, is attached below.

***Note:*** Rose plot representation helps to identify tissue drifts during time-lapse imaging.

Python code for rose plot representation:

import matplotlib.pyplot as plt

from mpl_toolkits import mplot3d

import numpy as np

import math

fig = plt.figure()

ax = plt.axes(projection = '3d')

ax.set_xlabel('x/μm')

ax.set_ylabel('y/μm')

ax.set_zlabel('z/μm')

ax.set_xlim(-50,50)

ax.set_ylim(-50,50)

ax.set_zlim(-50,50)

f = open('file_name.txt','r')

l = []

l = [line.split() for line in f]

l=np.array(l)

ll = np.transpose(l)

# Adapt to the column number in your file

x = ll[0]

x = x.astype(np.float)

y = ll[1]

y = y.astype(np.float)

z = ll[2]

z = z.astype(np.float)

t = ll[6]

t = t.astype(np.float)

num = ll[8]

num = num.astype(np.int)

position = x

position = np.c_[position, y]

position = np.c_[position, z]

ind_list = [0]

for i in range(1,len(num)):

 if num[i] == num[i-1]:

  j = i + 1

 else:

  ind_list = np.append(ind_list, j)

ind_list = ind_list.astype(np.int)

for i in range(1,len(ind_list)):

 k = ind_list[i-1]

 track = position[k] - position[k]

 a = ind_list[i] - ind_list[i-1]

 time = t[k] - t[k]

 if a > 1:

  for j in range(a):

   track = np.c_[track, position[k+j] - position[k]]

   time = np.append(time, t[k+j] - t[k])

  if len(track[0]) > 1:

   ax.plot3D(track[0],track[1],track[2])

14. Statistical analysis of B and plasma cells’ motility parameters.
  a. Import files containing displacement, track length and track duration of all cells belonging to the two B lineage cell populations in Origin (Origin Lab).
  b. Calculate average velocity, i.e. track length divided by track duration, and displacement rate, i.e. displacement divided by track duration and perform statistical analysis.

***Note:*** Alternatively, Excel, Python or MatLab routines can be used.

## Expected outcomes

**Figure 6:**
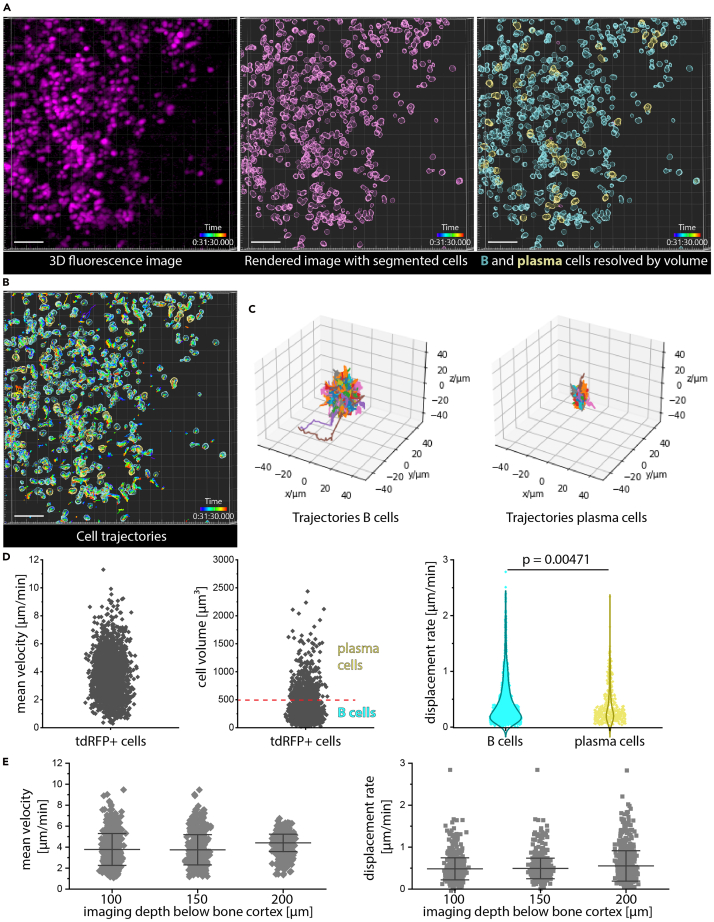
Analysis of B lymphocyte dynamics from three-photon microscopy data (A) Rendering of a 3D fluorescence image acquired in the tibia marrow of a CD19:LSL-tdRFP mouse (left). Denoising was performed using 2D N2V. Rendered image of segmented tdRFP^+^ cells (magenta) in Imaris, i.e. B lineage cells (middle image). Rendered image of B (cyan) and plasma cells (yellow), distinguished based on their volume in Imaris. Volume of B cells between 65 and 499 μm^3^; volume of plasma cells between 500 and 4189 μm^3^. (B) Rendered 3D image and tracks of segmented B and plasma cells analyzed from time-lapse 3D image data acquired every 30 s, over 60 min. (C) Rose plots of B cells (left) and plasma cells (right) showing the cell trajectories shown in B, all with starting point at origin (0,0,0). (D) Distributions of mean velocity of all tdRFP+ cells in B (left graph), of their cell volume (middle graph), and of displacement rate of B and plasma cells, respectively (right graph). B and plasma cells are resolved based on their volume, the threshold of 500 μm^3^ being indicated by the dashed red line in the middle graph. (E) Distributions of mean velocity (left graph) and of mean displacement rate (right graph) of all tdRFP+ cells, i.e. B lymphocytes, in three different depths in the tibial marrow, through intact bone cortex of 180 μm thickness. Both mean velocity and mean displacement rate do not depend on imaging depth. Whiskers in both graphs show SD and mean values. ANOVA one-way test was used for statistical analysis. Scale bar: 50 μm.

Exemplary time-lapse 3D images of B lineage cells in the tibia marrow acquired by *in vivo* 3PM at 1650 nm and 3 MHz repetition rate are shown in Methods video S1. The data are de-noised using the N2V trained algorithm on 3D data (400 × 400 × 41 μm^3^ data in CD19:LSL-tdRFP mice). tdRFP+ B lineage cells were segmented and tracked as described, results of segmentation and tracking being shown in Figure 6. B cells and plasma cells were distinguished by volume, as previously shown and confirmed in Blimp1:GFP mice^25^ (cells with a volume of 65 to 500 μm^3^ are classified as B cells, those with a volume between 500 and 4100 μm^3^ as plasma cells). From these data, rose plots showing whether the directed motion of the cells is random and which cell type displaces more are created. Additionally, statistics on mean velocity and displacement rate for the two cell types are generated, as shown in Figure 6. As expected, B cells have a higher average velocity and displacement rate than plasma cells. The values correspond to results obtained by 2PM in the tibia marrow,^25^ through mechanically thinned bone cortex. We found no dependence on imaging depth within the tibial marrow through intact bone cortex (180 μm thick) neither for the mean velocity nor for the mean displacement rate of B lymphocytes in CD19:LSL-tdRFP mice (Figure 6).

Methods video S1. *In vivo* 3D time-lapse video of tdRFP^+^ B lymphocytes (magenta) in the tibia marrow of a CD19:LSL-tdRFP mouse, related to Figure 6

## Quantification and statistical analysis

Statistical analysis of cell volume, displacement rate and mean velocity of B cells vs. plasma cells is described in detail in Rakhymzhan et al, 2024 and has been performed in Origin 2023.

## Limitations

The present protocol for *in vivo* 3PM deep-marrow cavity imaging in intact long bones retains great potential for extensive analysis of bone biology. Still, a few limiting issues need to be tackled in the future. Radiation at 1650 nm is not easily compatible with the excitation of GFP and its variants, typically used for fluorescent reporter mice, but rather approx. 1620 nm excitation.^16^ Therefore four-photon excitation (4P) is required. While reported,^13^^,^^16^^,^^26^ 4P excitation implies much higher photon flux densities and, by that, much higher pulse energies, increasing the risk of tissue photo damage, and requiring even narrower laser pulses.

Scattering of radiation at 1650 nm in bone tissue still leads to power loss, limiting the imaging depth in intact bones, calling for even longer excitation wavelengths, in the fourth IR tissue window, at approx. 2200 nm. While it is known that typically male mice have thicker long bone cortexes than females,^27^ we didn’t systematically analyze this aspect. Moreover, water absorption due to rovibronic transitions, especially in soft tissues such as the bone marrow, becomes relevant for power loss above 900 nm. Thus, for optimum 3P imaging of long bones, the appropriate excitation wavelength, which balances scattering and absorption in both bone matrix and soft marrow, still needs to be found. The impact of wavefront distortions caused by the tissue itself rises in 3PM with increased signal detection in deep tissue layers, calling for the implementation of wavefront correction using adaptive optics. As wavefront sensing in tissue is challenging, especially senseless wavefront correction methods are needed. In the present protocol, we did not perform wavefront correction, which, however, would be beneficial also in long bone imaging, as demonstrated for brain cortex imaging.^9^

The photon flux density at the focal point, which determines the imaging depth in tissue, depends not only on the point spread function but also on the laser pulse shape and width. Femtosecond pulses are dramatically broadened in biological tissues due to dispersion and other optically non-linear effects such as self-phase modulation.^28^ Thus, pulse compression for dispersion correction adapted to each tissue type and depth is expected to be beneficial. Due to complex, still pending developments, we haven’t implemented such adapted tissue dispersion correction in our system yet.

Several photophysical processes observed by 3PM take place on the scale of femtoseconds to few nanoseconds, e.g. fluorescence and higher-harmonics generation processes. The low laser repetition rates, needed to generate high pulse energy in 3PM, lead to time periods between consecutive pulses of hundreds of nanoseconds to microseconds. Thus, for the above-mentioned photophysical processes, the detectors collect noise, but no signal, over long periods of time. Therefore, detector time-gating is desirable to reduce noise in the generated images, but it was not used in this study. As we plan to detect with our 3PM system also photophysical processes on the microseconds scale, e.g. phosphorescence, we need to use dynamically adaptable time-gating to fit the timescales of all photophysical processes, which still needs to be developed and implemented.

## Troubleshooting

### Problem 1

No signal is detected underneath the bone cortex, neither fluorescence nor higher harmonics (method step 3e).

As three-photon processes are extremely improbable processes, delivering sufficient photon flux density at the imaged site is crucial. To avoid photo damage, increasing average laser power must be avoided.

### Potential solution

• Check the pulse width under the objective lens and minimize it by shifting the ZnSe wedge into or out of the beam path thus compensating the dispersion of the optical system. In this way, for a constant pulse energy, the peak energy and photon flux are increased.
• The laser beam is misaligned, and the effective NA is lower than the nominal NA of the objective lens. Adjust the beam expander to achieve 1.5× illumination of the back aperture of the objective lens.

### Problem 2

The z-position of the selected 3D stack imaged over time is constantly shifting (method step 6).

### Potential solution

Improve fixation of the tibia to reduce the effect of muscle contractions (oscillating movement) or relaxation (sinking). Restart a new measurement when z-drift is controlled.

### Problem 3

Fluorescence signal in the image is noisy and the cells can barely be detected (method step 4).

### Potential solution

Reduce the acquisition frequency, i.e. typically 800 Hz line frequency. A reduction to 400 Hz line frequency increases the signal.

***Alternatives:*** Use time gating for detection to reduce noise collection within the time periods, in which no signal is expected.

### Problem 4

The power is potentially too high. How to detect tissue damage? (method step 3).

### Potential solution

Test different power levels of the laser for the organ of interest by repetitive imaging over the time window you intend to perform the experiment. Cell motility and blood flow rate slow down are indicators of tissue disturbance, whereas interrupted blood flow and the appearance of micro-plasma in the tissue are clear signs of tissue damage.

***Alternatives:*** Tissue damage can be further confirmed by immunofluorescence histological analysis of the paraformaldehyde fixed tibia slices, after 3PM imaging, on which heat shock protein (HSP70), apoptosis markers, such as TUNEL, and neutrophil granulocytes (Lys6C) are labeled.

## Resource availability

### Lead contact

Raluca A. Niesner, raluca.niesner@fu-berlin.de.

### Technical contact

Asylkhan Rakhymzhan, asylkhan.rakhymzhan@gmail.com.

### Materials availability

• This study did not generate new unique reagents.

### Data and code availability

• Original/source data for Figure 6 in the paper is available on Zenodo: https://doi.org/10.5281/zenodo.8383833.
• The datasets generated during this study are available at Zenodo: https://doi.org/10.5281/zenodo.8383833.
• The published article includes all data and code generated or analyzed during this study.

## Acknowledgments

We acknowledge the 10.13039/501100001659German Research Foundation for financial support under grant CRC1444 (427826188), P14, and FOR5560 (505372148), P2, to R.A.N. and A.E.H. as well as HA5354/12-1 (511083451) to A.E.H.

## Author contributions

A.R. integrated the optical parametric amplifier. A.B. integrated the ZnSe-based pulse compression and the second-order interferometric autocorrelator in the upright microscope. A.F.F. and A.B. performed validation experiments and analyzed data. A.R. and R.A.N. performed validation and *in vivo* imaging experiments and analyzed data. R.G. performed the mouse surgery for imaging. I.B. contributed to data analysis. R.A.N. and A.E.H. designed the study, supervised the project, and curated the data. A.B., A.R., and R.A.N. wrote the protocol and made figures.

## Declaration of interests

The authors declare no competing interests.
